# Child Abuse Potential in Young German Parents: Predictors, Associations with Self-reported Maltreatment and Intervention Use

**DOI:** 10.1007/s10578-021-01157-y

**Published:** 2021-03-17

**Authors:** Katrin Lang, Christoph Liel, Ulrike Lux, Heinz Kindler, Marc Vierhaus, Andreas Eickhorst

**Affiliations:** 1 Research Department Early Childhood Intervention, German Youth Institute, Nockherstraße 2, 81541 Munich, Germany; 2 Child Guidance Center Ingolstadt, Gabelsberger Straße 46, 85057 Ingolstadt, Germany; 3 Education and School Counselling District Gütersloh, Herzebrocker Str. 140, 33334 Gütersloh, Germany; 4 Faculty of Social Work, University of Applied Sciences and Arts Hannover, Blumhardtstraße 2, 30625 Hannover, Germany

**Keywords:** Child abuse potential, Parental risk factors, Parenting stress, Early childhood, Early childhood intervention

## Abstract

Since child maltreatment has highly negative effects on child adjustment, early identification of at-risk families is important. This study focuses on longitudinal risk factors for child maltreatment and associations between abuse risk and occurrence. It also examines whether abuse risk and involvement in early childhood intervention are associated. The sample comprises 197 German caregivers with children under 3 years of age. Data was collected in two waves. The Brief Child Abuse Potential Inventory assessed abuse risk. Socio-demographic, parent, child and family-related risk factors were measured using screening tools. The analysis revealed that parental characteristics (psychopathology, own maltreatment experiences etc.) were associated with concurrent abuse risk. Longitudinal changes in abuse risk were linked to caregiver education and child-related factors. Cumulative risk did not explain more variance than specific risk factors. Significant associations with caregiver-reported abuse were found, and data suggest that some burdened families cannot be reached by early childhood intervention.

## Introduction

According to official statistics, in 2019 55,527 children were victims of substantiated maltreatment in Germany [1]. Infants and toddlers constitute a large part of this group: 20.7% of children with substantiated maltreatment were under the age of 3 years (*N* = 11,517) and children under the age of 1 year had the highest prevalence of victimization (8.3%). Neglect was recorded in 67.27% of substantiated cases among children under three. Physical maltreatment was documented in 20.11%, psychological maltreatment in 28.74%, and sexual abuse in 1.46% of cases [1]. These numbers are more than alarming, especially given that each year many cases remain unreported. Yet, they align with findings from other national and international studies investigating the frequency of different types of abuse, with neglect representing the most frequent type of maltreatment [2].

Over the last decades, research that has focused on the consequences of child maltreatment, reported numerous negative effects on physical, cognitive, psychological and social outcomes [3]. Especially victims of chronic maltreatment in infancy and toddlerhood [4], and children exposed to various types of maltreatment [5, 6] were found to experience substantial negative outcomes. Therefore–to protect children from harm–the early identification of families at (high) risk for child maltreatment is an important goal in child protection. Another key objective is to reach families at risk and motivate them to participate in targeted prevention services, because we know that parenting programs and other early childhood intervention are able to reduce child maltreatment (e.g. [7]). Consequently, in the current study, we aim to examine the links of being at risk of perpetrating child maltreatment, and actual incidents of family violence. Also its association with the use of early childhood intervention will be explored.

### Risk Factors for Child Abuse

Serious problems in parenting and child abuse occur disproportionately often in families exposed to a higher number of risk factors [8, 9]. Prior research suggests that child maltreatment is a complex phenomenon caused by an interaction of multiple factors that simultaneously impact parents [10]. According to the developmental-ecological theory of abuse [11], risk factors are located on different system levels, ranging from exerting direct influence on child adjustment (*proximal risk factors*, i.e. parenting) to having more indirect effects (*distal risk factors*, i.e. poverty). Recent research focuses on the identification of critical risk factors for child abuse and neglect with the aim of identifying families at risk for child maltreatment at an early stage. One of the most comprehensive studies on this topic is the meta-analysis by Stith and colleagues [9]. This meta-analysis comprised 155 studies between 1969 and 2003, and included 39 risk factors related to physical abuse and neglect. The largest effect sizes regarding abuse were found for parental anger, the perception of the child as a problem as well as for high conflict and low family cohesion. Also, an unplanned pregnancy or lack of social support, the child’s lower social competence and low SES showed at least a medium effect size. Not least parental characteristics such as anxiety, depression, low self-esteem, life and parenting stress were significant risk factors. In Germany, only two older small-scale studies could be identified, which addressed risk factors for child abuse and neglect [12, 13]. Hence, current data on risk factors for child maltreatment, particularly in Germany are missing.

Besides findings on the significance of single risk factors, a growing number of studies highlights the role of the combination of risk factors in predicting child abuse [14–16]. There is an ongoing discussion on the extent to which some risk factors weigh more than others. Also the large variance of effect sizes in the meta-analysis of Stith et al. [9] points in this direction. However, previous evidence suggests that it is the accumulation of risk, which is particularly detrimental to child welfare, rather than any specific factor. In one study, the total number of risk factors was shown to be the most reliable predictor for substantiating a case of assumed neglect or abuse [15], and in another, children who were affected by four out of five relevant risk factors were found to have a seven-fold higher risk of being abused [16]. On this basis, the current study takes into account the diversity of significant risk factors and seeks to examine, whether the accumulation or any specific combination of risk factors might be more relevant to predict child maltreatment in families with young children.

### Measurement of Child Abuse and Child Abuse Potential

Child abuse may be assessed in retrospect via self-report on childhood experiences, through parents’ self-report on their behavior towards children, sentinel report or official statistics. These methods often provide different results. Single informant reports, and official statistics generally underestimate the prevalence of child abuse [17, 18]. In addition, precursors, which accumulate to a higher risk for actual acts, are more important than child abuse itself for prevention purpose. Yet, measures for testing participant eligibility were needed here. As a result, instruments measuring risk for child maltreatment were developed. Such instruments assess the probability that a respondent will become a perpetrator. Internationally, the most commonly used instrument for this purpose is the Child Abuse Potential Inventory CAP [19], the current “gold standard” in the assessment of child abuse risk [20]. The CAP seems able to successfully distinguish between maltreating and non-maltreating parents, and to predict subsequent incidents of abuse effectively [19, 21]. Since the CAP contains correlates of maltreatment, but does not ask directly for acts of abuse or neglect, it has a higher acceptability among parents. Nevertheless, containing 160 items, the CAP is a costly and time-consuming measure inappropriate for screening and survey purposes. To address this challenge, a short form of the Child Abuse Potential inventory, a brief CAP was developed BCAP [22]. It contains 33 items, which makes it a less burdensome instrument in evaluative contexts. Besides the validation study, there are already some studies, which examined the applicability and validity of the BCAP outside the US with satisfactory results, at least for mothers [23, 24]. However, there is a discussion on the predictive value of risk assessments, with a recent meta-analysis certifying the BCAP a below average predictive accuracy [25]. Accordingly, it is particularly important to shed light on the links between child abuse potential and risk factors for child maltreatment in order to identify burdened families more accurately.

### Aim of the Study

The current study thus addresses four research questions on child abuse potential in German families with young children and different levels of risk. First, we will investigate the associations between different socio-demographic or psychosocial risk factors and child abuse risk cross-sectionally and longitudinally. According to Stith et al. [9], we expect moderate to high associations. Second, we will examine, whether there are specific combinations of risk factors with high predictive power and/or if child abuse risk (BCAP) increases with increasing number of risk factors. Furthermore, regarding the predictive value of the BCAP, we want to know if, as expected, child abuse risk is higher among parents who reported any events of family violence. Finally, we will explore the links between child abuse risk and use of universal or targeted prevention. Here we expect at least a moderate association, since one main goal of early childhood intervention is to reach families at risk.

## Method

### Sample

The current study is part of a series of studies named KiD 0–3 (“Children in Germany aged 0–3”) [26]. At first, two *pilot studies* that took place between October 2013 and February 2014 in two major German cities, aimed to provide preliminary prevalence rates of risk factors and test different recruitment strategies of families with infants and toddlers (*N* = 6000). Ethical approval for the pilot studies was granted by General Medical Council in the North-Rhine region (No. 2013247). In order to follow families with different levels of psychosocial burden longitudinally using an extensive assessment battery [27], a small sub-sample of the pilot studies (*N* = 197, with an oversampling of burdened families) was recruited for an *in-depth study.* Therefore, families were recruited out of all families in the pilot studies who agreed to participate in further assessments and whose child grew into one of the targeted age cohorts (10–14 months or 17–21 months) during the inquiry period (*N* = 937). To achieve a rather equal distribution of differently burdened families [27], these families were grouped into families of low (0–1 risk factor), medium (2–3 risk factors) and high psychosocial burden (4 and more risk factors) according to the number of distal and proximal risk factors in the pilot studies’ screening Table 1. Distal risk factors included social welfare receipt, low level of education, parental history of child abuse or neglect, and substance abuse amongst others. Proximal risk factors included e.g. interparental conflict, domestic violence, maternal depression, and the child’s negative affectivity. In case one family refused to participate, another randomly selected family with the same level of burden was contacted and—provided they agreed—included in the study until the targeted sample size was reached. Families with a high psychosocial burden accounted for 26.4% of the sample with a mean of 5.44 risk factors, those with medium burden with an average of 2.30 risk factors accounted for 36.0% of the sample. 37.6% of the families had a low burden with, on average, 0.54 risk factors. The KiD 0–3 *in-depth study* was conducted in accordance with the Code of Ethics of the World Medical Association (Declaration of Helsinki).

**Table 1:** Categorization of risk factors

Risk factor	Risk	No risk
1. Parent education	ISCED low	ISCED medium/high
2. Parent immigrant background	Yes	No
3. Social welfare receipt	Yes	No
4. EBI parent domain	≥ 80	< 80
5. EBI child domain	≥ 50	< 50
6. Stress (PSS-4)*	≥ 14	< 14
7. Lack of social support*	≥ 4	< 4
8. Partnership dissatisfaction (DAS)	≥ 9	< 9
9. Self-efficacy in parenting (SENR)*	≤ 79	> 79
10. Strain due to child regulatory problems*	≥ 4	< 4
11. Anger*	≥ 2 items yes	< 2 items yes
12. Depression/anxiety (PHQ-4)	≥ 6	< 6
13. Adverse childhood experiences (ACE)	≥ 4	< 4

*No author information on cut-offs, 1 SD rule

Overall, 197 children and their caregivers (98 in cohort 1, 99 in cohort 2) participated in the in-depth study. At the first assessment (T1), the mean age was 11.8 months (cohort 1) and 18.7 months (cohort 2) respectively. The second data assessment (T2) occurred seven months later on average. Half (*n* = 99) of the children in the sample were male. In 191 of the cases (97%), the primary caregiver was the mother. Primary caregivers had a mean age of 34.19 years (*SD* = 5.51, *Min* = 21, *Max* = 65). 9 of 10 caregivers cohabited with the other biological parent. 26% of primary caregivers had an immigrant background, and 16% received social welfare. Based on the standard classification for educational attainment (ISCED), parents’ education was grouped into three broad categories: 11% had a low, 36% had a medium, and 53% a high level of education. At T2, there was a systematic dropout (*n* = 15, 7.6%), particularly among those with a low level of education [27].

### Procedure

Data were collected during semi-structured home visits including observations, questionnaire assessments and experimental tests. Participants received small financial incentives (€40 per family). The researchers in contact with participants were not informed of the family’s risk group categorization to ensure independent examination. The current study comprises data from the primary caregiver focusing on child maltreatment and psychosocial risk, which was only available via self-report questionnaires, collected mainly during the home visit (some information stem from the pilot studies).

### Measures

#### Brief Child Abuse Potential

Child abuse potential was assessed with the brief form of the Child Abuse Potential Inventory (CAP, [21]), the BCAP [22]. The BCAP comprises 33 items with a 24-item maltreatment scale and a nine-item validity scale (containing a lie scale and a random response scale) in an “agree/disagree” format. With a cut-off of nine for caregivers at risk, sensitivity and specificity was 0.93 each. A cut-off of 12 yielded sensitivity and specificity values of 0.91 and 0.93. In this study Cronbach’s alpha revealed to be *α* = 0.86 at T1 and *α* = 0.88 at T2.With *r* = 0.77 test–retest reliability was acceptable.

#### Socio-Demographic Factors

Information of both parents on immigrant background, level of education and receipt of social welfare was assessed during the pilot study. Immigrant background was based on §6 of the German MighEV act: not having a German nationality or being a first generation immigrant or having at least one parent being born outside of Germany. As an indicator for poverty, families were asked whether they were dependent on social welfare receipt (“ALG II” or other). Other demographic information on parental and child age, gender and relationship status was provided by both caregivers.

#### Parenting Stress

To assess parenting stress, an adapted German version of the Parenting Stress Index (German: EBI) [28] was used. The instrument comprised 48 items measuring parenting stress in two domains: (1) demanding characteristics and behaviors of the child (child domain with five sub-scales, e.g., distractability/hyperactivity, demandingness), and (2) restrictions in parenting functions (parent domain with seven sub-scales, e.g. competence, role restriction). Underlying data from T1 of this study resulted ininternal consistencies of *α* = 0.92 (parent domain, 28 items) and *α* = 0.88 (child domain, 20 items).

#### Life Stress

Life Stress was assessed by a four-item short form of the Perceived Stress Scale (PSS-4), which was comprehensively validated and utilized in several surveys (e.g. [29]). In contrast with the original five-point scale, the PSS in this study used a six-point scale with an additional response option (“always”). Data resulted in a very good internal consistency of α = 0.80 at T1.

#### Lack of Social Support

To assess social support, two items were drawn from the German Family Panel pairfam [30]. The items assessed the perceived availability of social support concerning care and advice. In a change to the original scaling, a four-point scale ranging from “absolutely true” to “not at all true” was used. Higher scores indicated lower social support. The scale had an acceptable internal consistency of α = 0.66 at T1.

#### Partnership Dissatisfaction

To assess partnership quality, a German short version of the Dyadic Adjustment Scale (DAS-4; [31]) was used. Three items are rated on a six-point scale from “never” to “always”. The fourth item concerns the overall satisfaction with the partnership and is rated on a seven-point scale from “extremely unhappy” to “perfect”. DAS-4 scores range from 0 to 21 with higher scores indicating lower partnership satisfaction in this study. With *α* = 0.62 at T1, Cronbach’s alpha was acceptable in the current study.

#### Self-efficacy in Parenting

To assess parental self-efficacy, a German translation of the postnatal Parenting Self-Efficacy in Nurturing Role Questionnaire (SENR; [32]) was used. The questionnaire assesses parental attitudes concerning parenting competence. It consists of 16 items rated on a seven-point Likert scale from “not at all representative of me” to “strongly representative of me". Item scores were added to build a sum-score. The SENR showed good internal consistency of *α* = 0.77 at T1.

#### Strain due to Child Regulatory Problems

Three questions were developed assessing parental psychological strain due to infant regulatory problems such as excessive crying, feeding and sleeping problems. Strain was rated for each area of regulation on a four-point scale from 0 “not at all” to 3 “very much”. A sum-score varied from 0 to 9.

#### Anger

As there is no brief screening to assess parental anger, two items from the CAP [21] were used, which are not part of the BCAP [22]. They describe a general tendency to get angry easily. Like in the CAP, the format in this study was dichotomous (“agree/disagree”).

#### Depression/Anxiety

To assess psychiatric symptoms, a short form of the Patient Health Questionnaire (PHQ-4; [33]), a widely used and internationally validated instrument, was administered. This four-item self-report questionnaire consists of two two-item sub-scales measuring core symptoms of depressive or generalized anxiety disorders. The responses to items ranged from 0 “never” to 3 “every day”. PHQ-4 scores ranged between 0 and 12 and the full scale showed a good internal consistency of α = 0.78 at T1. A PHQ-4 total score ≥ 6 has been recommended as an indicator of the presence of a depressive or an anxiety disorder [34].

#### Adverse Childhood Experiences

To assess parents ‘own maltreatment experiences, the German Adverse Childhood Experience Questionnaire (ACE; [35]) with satisfactory psychometric properties was used. Negative experiences were summed up from 0 to 10.

#### Family Violence

A six items-scale assessed child abuse, neglect and exposure to domestic violence since childbirth, adapted from the Juvenile Victimization Questionnaire [36]. Because research shows that witnessing domestic violence has negative effects on child development equal to the effects of physical abuse victimization [37], we defined exposure to domestic violence as a form of psychological maltreatment corresponding to previous research [38]. Hence, we assessed three items addressing child maltreatment (physical abuse, neglect, shaking), and three items addressing domestic violence (threat, damage caused by argument, domestic abuse). All children who were reported to have experienced at least one of the six abusive behaviors were categorized as having suffered from family violence.

#### Use of Early Childhood Intervention

Participants were asked whether they had been involved in any universal or targeted prevention services. Universal prevention services included parent–child courses, feel-well interventions, volunteer visits, or telephone advice. Targeted and indicated prevention services included specialized counseling (e.g. for excessive crying), home visiting programs, child guidance counseling or support services provided by child welfare services. Parents who reported use of at least one prevention service were categorized as having used early childhood intervention.

If not stated otherwise by the authors of the respective scales, a maximum of 10% of missing values per scale was accepted. Missing data were imputed using mean scores of other items of the scale.

## Results

### Descriptive Statistics of the Brief Child Abuse Potential Inventory

At T1, the BCAP inventory had a mean score of 4.38 (*N* = 192, *SD* = 4.35; α = 0.86) for main caregivers. At T2, the mean score was 4.01 (*N* = 176, *SD* = 4.31; α = 0.88) with a medium to high stability of *r* = 0.77, *p* < 0.001. Overall, there was no change in mean abuse risk across time. While parents of children in the younger age group had marginally significant higher abuse scores at T1, *t*(181.83) = − 1.66, *p* = 0.10, *d* = 0.24, parents in both child age groups did not differ in abuse risk at T2. Using the cutoffs established by Ondersma et al. [22], there were 6.6% at T1 and 7.1% at T2 (cut off 12) of subjects who have a risk for child abuse. Using the cut-off of 9, these percentages were 12.7% at T1 and 10.2% at T2. Further information concerning validity and factor structure of the BCAP in the current sample are presented elsewhere [24].

Applying the guidelines regarding the validity scale established by Walker and Davies [23] and the suggestion of Milner [19], there were 25.9% (*n* = 51) invalid protocols at T1 and 17.3% (*n* = 34) at T2. Since removing these individuals would have resulted in a substantially reduced sample—even after retaining the participants with BCAP risk scores > 12 as recommended by Milner [19], further analyses were performed with this subsample.

Participants with invalid protocols scored significantly higher in all relevant risk factors as well as in BCAP abuse risk, *t*(189) = − 4.54, *p* < 0.001, *d* = 0.86 before retaining the highly at-risk group (BCAP risk > 12), and they did not differ or still revealed to be more burdened after retaining the high scoring BCAP-group. They were also more likely to have an immigrant background with *χ*^2^(1, 192) = 5.56, *p* = 0.02. Furthermore, the items on the lie-scale were ticked by up to 63.5% of participants, one item of the random-response-scale even by 90.4% of participants see discussion in [23], and BCAP risk score was positively linked to the validity scales (lie scale *r*_*T1*_ = 0.45, *p* < 0.001, random response scale *r*_*T1*_ = 0.21, *p* = 0.004).

Thus, participants with invalid protocols seemed not to succeed in ‘faking good’, even more so they tended to be more at-risk. Therefore an exclusion of these protocols seemed to restrict the sample to less burdened families and families without immigrant background. This would limit the variance of the sample. Consequently, and because of the ongoing discussion about meaning and function of the validity scale see [23, 24], we decided on a different approach by including all protocols at first, and doing each analysis with the full sample and without the invalid protocols (cross-check). It is stated if using only valid protocols revealed different results.

### Associations Between Risk Factors and Child Abuse Potential

At first, associations between risk factors at T1 and BCAP inventory scores at T1 and T2 (Table 2) were examined. All risk factors except immigrant background were significantly associated with the BCAP abuse scale. Multiple linear regressions were calculated to predict child abuse potential based on the 12 significantly associated risk factors Table 3, top). Risk factors explained 64% of total variance in T1 child abuse potential, *F*(12, 142) = 23.78, *p* < 0.001. Parenting stress in the parent domain, partnership dissatisfaction, anger, depression/anxiety and maternal ACEs significantly added to explained variance. When child abuse potential at T1 was controlled, risk factors explained an additional 10% of variance in T2 abuse potential. Seven months later, still 62% of the total variance in T2 abuse risk was explained by the risk factors, *F*(13, 138) = 20.12, *p* < 0.001. Here, level of education, life stress, parenting stress in the child domain, strain due to child regulatory problems and also maternal ACEs added significantly to explained variance.

**Table 2:** Association of risk factors at T1 with the BCAP inventory score

	BCAP inventory score	
	T1	T2
1. Low parent education	− 0.23**	− 0.26**
2. Parent immigrant background	0.10	0.03
3. Social welfare receipt	0.21**	0.20**
4. EBI parent domain	0.52***	0.48***
5. EBI child domain	0.31***	0.37***
6. Stress (PSS-4)	0.61***	0.60***
7. Lack of social support	0.28***	0.30***
8. Partnership dissatisfaction (DAS)	0.46***	0.45***
9. Self-efficacy in parenting (SENR)	− 0.49***	− 0.41***
10. Strain due to child regulatory problems	0.36***	0.22**
11. Anger	0.67***	0.57***
12. Depression/anxiety (PHQ-4)	0.69***	0.55***
13. Adverse childhood experiences (ACE)	0.30***	0.33***

*N*_*T1*_ = 173–192 and *N*_*T2*_ = 167–176

***p < 0.001, **p < 0.01, *p < 0.05

**Table 3:** Regression models predicting child abuse potential

Predictors	BCAP T1			BCAP T2		
	*B*	*SE B*	*ß*	*B*	*SE B*	*ß*
Linear regression with all 12 significant risk factors						
BCAP T1 (Δ*R*^*2*^ = 0.56)	–	–	–	0.82	0.06	0.75***
Parent education	− 0.16	0.36	− 0.04	− 1.22	0.41	− 0.18**
Social welfare receipt	− 0.30	0.68	− 0.02	− 0.17	0.75	− 0.01
EBI parent domain	0.04	0.02	0.21*	0.02	0.02	0.07
EBI child domain	− 0.03	0.02	− 0.09	0.05	0.03	0.14*
Stress (PSS-4)	0.05	0.09	0.04	0.24	0.10	0.20*
Lack of social support	− 0.20	0.16	− 0.08	− 0.07	0.18	− 0.03
Partnership dissatisfaction (DAS)	0.26	0.09	0.17**	0.09	0.10	0.06
Self-efficacy in parenting (SENR)	− 0.03	0.02	− 0.09	0.03	0.03	0.08
Strain due to child regulatory problems	− 0.09	0.18	− 0.03	− 0.34	0.20	− 0.11^+^
Anger	1.58	0.34	0.30***	0.16	0.41	0.03
Depression/Anxiety (PHQ-4)	0.67	0.13	0.34***	− 0.09	0.16	− 0.04
Adverse childhood exp. (ACE)	0.22	0.10	0.12*	0.20	0.11	0.10^+^
Total *R*^*2*^(n)	0.64*** (154)			0.62*** (151)		

	BCAP T1		BCAP T2		
	Δ *R*^2^	*ß*		Δ*R*^2^	*ß*
Stepwise regression					
			Control BCAP T1	0.56***	0.75***
Step1	0.47***		Step1	0.04***	
PHQ-4		0.69***	Parent educat		− 0.19***
Step 2	0.10***		Step 2	0.03***	
Anger		0.38***	Stress		0.21**
Step 3	0.05***				
EBI parent domain		0.27***			
Step 4	0.02**				
DAS		0.17**			
Total *R*^*2*^(n)	0.64*** (154)		Total *R*^*2*^(n)	0.62*** (151)	

***p < 0.001, **p < 0.01, *p < 0.05, ^+^*p* < 0.10

### Prediction of Child Abuse Potential by Specific Combinations and Number of Risk Factors

Regarding our second research question, whether there were specific combinations of risk factors explaining maximal variance, stepwise regression analysis was conducted Table 3, bottom). At T1, the final model predicted 64% of variance, *F*(4, 150) = 68.79, *p* < 0.001. It included the following risk factors: depression/anxiety, anger, EBI parent domain and partnership dissatisfaction. For T2, the stepwise regression model explained 62% of variance after controlling for T1 BCAP inventory score, *F*(3, 148) = 81.50, *p* < 0.001. Level of education and life stress were revealed to significantly added to explained total variance.

To analyze group differences in longitudinal development of abuse risk, repeated measures of analysis of variance (ANOVA) were used. There was a significant interaction between time (T1 versus T2) and level of education (low versus medium/high), *F*(1, 173) = 5.18, *p* = 0.024. While BCAP scores increased among parents in the group with low education, they decreased among the groups with medium to high level of education. Another marginally significant interaction emerged between low/medium vs. high stress (cut-off 16) and time (T1 versus T2), *F*(1, 173) = 3.64, *p* = 0.058: child abuse risk decreased among groups with low levels of stress at T1, but increased—although both non-significantly—among groups with a higher stress level at T1. For strain due to child’s regulatory problems, we found a marginally significant time (T1 versus T2) x regulation problems (low versus high) interaction, *F*(1, 169) = 3.21, *p* = 0.075. While child abuse risk was stable among caregivers who did not report strain due to child’s regulatory problems, it decreased slightly among parents who reported respective strain at T1. No interactions were found for the EBI child domain. Between subject effects were significant in all analyses.

The valid BCAP protocols revealed the same results in both linear and stepwise regression for T1 abuse risk. When T2 abuse risk was predicted for valid protocols only, level of education and maternal ACEs were revealed to be significant predictors.

To answer the question whether the accumulation of risks is more predictive for child abuse potential, an additive risk index was built. All risk factors were dichotomized (present/not present). Table 1 shows the respective classification scheme. Where authors did not provide information regarding cut-offs, scores of ± 1SD were categorized to be at-risk. Information on all 13 risk factors was available from 160 participants. The average level of risk factors was 1.82 (*SD* = 1.97, *Min* = 1, *Max* = 9). The correlation between the number of risk factors and BCAP inventory score was *r* = 0.71, *p* < 0.001 at T1 and *r* = 0.64, *p* < 0.001 at T2. The number of risk factors present differed significantly between the groups with (cutoff 9, *M* = 4.82; *SD* = 2.04) and without child abuse risk (*M* = 1.45; *SD* = 1.64), *t*(157) = − 7.80, *p* < 0.001, *d* = 2.01, at T1. Similar difference between the risk (*M* = 4.87; *SD* = 2.00) and non-risk group (*M* = 1.47; *SD* = 1.66) was found at T2, *t*(155) = − 7.38, *p* < 0.001, *d* = 2.02. When the number of risk factors was entered as predictor, it explained 50% of variance in T1 child abuse risk, *F*(1,157) = 158.29, *p* < 0.001. After controlling for T1 abuse risk, the number of risk factors significantly explained an additional 3% of the variance in T2 child abuse risk. *F*(2,153) = 99.03, *p* < 0.001, *R*^*2*^ = 0.56.

### Accordance Between Child Abuse Potential and Actual Reports of Family Violence

The overall rate of family violence was low in the current sample: child maltreatment occurred in 8 (T1) or 2 families (T2), domestic violence in 29 (T1) or rather 26 families (T2). The association between BCAP scores and family violence (any item yes versus all no) was significant at T1 (*N* = 192, *r*_*pb*_ = 0.20, *p* = 0.004) and T2 (*N* = 175, *r*_*pb*_ = 0.34, *p* < 0.001). Partial correlations (control: family violence T1) revealed a significant association between BCAP score at T1 and the number of family violence incidents at T2 (*r* = 0.28, *p* < 0.001, *df* = 174). Similarly, a Chi-square test revealed a significant accordance between T1 abuse potential (high vs. low, cut-off 9) and family violence occurrence at T2, χ^2^(1) = 5.04, *p* = 0.037. This suggests that participants with higher BCAP scores were more likely to report incidents of family violence seven months later. Mean BCAP scores differed significantly between participants who reported any events of maltreatment or domestic violence, and participants who did not Table 4.

**Table 4:** Differences in BCAP abuse scale mean scores between parents with and without reported family violence and with or without use of universal or targeted prevention services

Group	N	M	SD	t-test	Cohen’s d
T1					
NSPCC					
Yes	25	6.68	4.91	*t* (190) = 2.88, *p* = 0.004	0.62
No	167	4.03	4.17		
T2					
NSPCC					
Yes	21	7.95	5.73	*t* (22.44) = 3.48, *p* = 0.002	1.11
No	155	3.47	3.80		
T1					
Universal					
Yes	147	3.90	3.99	*t* (178) = − 2.71, *p* = 0.007	0.52
No	33	6.09	5.07		
Targeted					
Yes	26	5.57	5.36	*t* (176) = 1.61, *p* = 0.108	0.34
No	152	4.11	4.06		
T2					
Universal					
Yes	139	3.63	3.97	*t* (21.50) = − 1.51, *p* = 0.145	0.49
No	20	5.70	5.95		
Targeted					
Yes	36	6.40	5.74	*t* (42.56) = 2.98, *p* = 0.005	0.72
No	139	3.41	3.64		

### Child Abuse Risk and Use of Universal and Targeted Prevention Services

Finally, we wanted to know if families with higher BCAP scores use early childhood intervention more often. To test whether there were differences in BCAP scores according to the type of intervention used, comparisons of mean scores for participants who used universal or targeted prevention services and those who did not were calculated Table 4. Universal prevention services were used more often by families with lower abuse risk, especially at T1, whereas families with child abuse risk at T2 more often used targeted prevention services. A Chi-square test showed that there was no significant accordance between use of targeted prevention services (yes/no) and child abuse potential (high/low, cut-off 9) at T1. Contrastingly, the accordance revealed to be significant at T2 with χ^2^(1) = 6.37, *p* = 0.012. This shows that parents with high child abuse risk used targeted prevention services more often. Using only valid protocols revealed similar, yet more prominent results.

## Discussion

The current study aimed to shed light on factors associated with the child abuse potential of German mothers with young children. We not only looked at the relative impact of various risk factors for child abuse risk in this sample, but also investigated whether family violence was linked to child abuse potential, and if it subsequently led to a higher use of early childhood intervention.

### Prediction of Child Abuse Risk by Single and Combined Risk Factors

Almost all risk factors were associated with the BCAP inventory abuse risk, at least to a moderate degree. The smallest associations were found for socio-demographic and child-related risk factors. As expected from the theoretical CAP framework, parents’ intrapersonal functioning and parents’ perceptions were found to be highly associated with child abuse risk assessed with the BCAP in this study, too. This was also seen in the regression models: The accumulation of all risk factors together explained the same variance in T1 child abuse risk as four specific variables combined focusing on (inter)parental characteristics (depression/anxiety, anger, parenting stress and partnership dissatisfaction). Therefore, the latter combination of risk factors seems a good estimate for child abuse risk in the current study. Although, the findings should be interpreted with respective caution due to the screening character of the measures, they are in line with previous evidence, which found a high risk of child abuse for parents with psychopathological symptoms, particularly co-morbid anxiety and depression [39, 40] or parental anger [9, 41, 42]. Even in terms of neglect, previous research has identified parental anger as a risk-heightening factor [8]. Also parental stress was identified to be a risk factor for child abuse in previous studies [42, 43].

The current study also revealed the importance of distinguishing between child- and parent-related subscales. In general, the results of this study show, that child-related risk factors do not seem as important as parent-related risk factors when looking at child abuse potential. This is particularly true for parenting stress. But whereas parenting stress in the parent domain predicted initial child abuse risk, parenting stress in the child domain was only moderately linked to child abuse potential. However, differences occurred when changes in child abuse risk were examined seven months later. Interestingly, while initially child abuse risk was predicted by parental characteristics only, change in abuse risk was associated with child factors and more distal risk factors. Although BCAP maltreatment risk was rather stable across time, analysis revealed that some factors influenced change in BCAP. Five factors individually added to explained variance in BCAP abuse scale changes: level of education, life stress and parenting stress in the child domain, strain due to the child’s regulatory problems, and parental history of childhood maltreatment. In particular, abuse potential increased when parents had a low level of education, higher levels of life stress or perceived their child to be problematic. This corresponds to the social information processing model proposed by Milner [44]: Highly stressed parents, parents with low levels of education or parents with negative attitudes towards their child might lack adaptive strategies and return to basic belief structures and consequent behaviors when faced with parenting challenges. Furthermore, parenting challenges often rise with a growing child [45] in general. For early intervention these parents who become overburdened by their developing–and thus more challenging–child are an important target group. Building up resources, networks and competences in these families at an early stage might hinder the increase in child abuse potential.

Partnership dissatisfaction was found to be the fourth most important predictor in the current study. While we just used a brief indicator of partnership satisfaction limiting the possibilities of interpretation, results parallel other findings showing that high levels of conflict, decreased parental cooperation or relationship dissatisfaction lead to increased personal stress, harsh discipline, and elevated abuse risk [46, 47]. In contrast, good couple functioning [48] and higher paternal involvement [49], which would also point to a supportive interparental relationship, seem to be protective factors, e.g. by reducing maternal child abuse risk. Altogether, these results suggest, that beyond being burdened by child characteristics, some internal factors in the parents themselves, e.g. parental personality or parents’ mental state increase the risk of perpetrating child maltreatment.

Furthermore, initial parental psychological strain due to reported infant regulatory problems was associated with a decrease in abuse risk. This was unexpected. As most children make great improvement in self-regulation during infancy and early childhood [50], in general, many parents’ frustration with the child’s regulatory problems might have decreased from T1 to T2. In particular regarding families who experienced a higher burden due to their children’s regulatory problems, any decrease in such problems should lead to a reduction in parents’ overextension and, thereby in the risk for child abuse.

### Cumulative Risk and Child Abuse Potential

This study showed that a cumulative risk index can also explain significant variance in child abuse risk. This fits with existing findings, demonstrating the significance of cumulative risk repeatedly [10, 15, 16].

Cumulative risk indices were used successfully in planning adequate interventions [5] and found to be better predictors for abuse risk than single risk factors [14]. Our findings link to a deeper understanding of abuse as a highly complex, social problem that may result from a variety of burdens [11, 41]. However, as discussed above, a particular combination of risk factors exceeded the predictive power of the cumulative risk index in the current sample. This finding suggests that practitioners, policy makers and researchers should focus on more than the mere number of risk factors present.

### Associations Between Abuse Risk and Parent-Reported Family Violence

There were significant associations between the BCAP abuse scale and parent-reported incidents of maltreatment and domestic violence. However, regarding the mere number of parent-reported child abuse and neglect, previous estimates of the prevalence of maltreatment [51] far exceeded underlying prevalence rates. This points to a high rate of unreported incidents. On the other hand, exposure to domestic violence seems to be much more common than child maltreatment itself, indicated by higher numbers of parent-reported domestic violence. This corresponds with the meta-analysis by Kindler [52], who found that 11.2% of parents indicated their children’s exposure to massive interparental quarrels or domestic violence. By contrast, only 3.1% of parents reported any kind of child maltreatment. Hence, even though more rarely, maltreatment seems to be perceived more severely in public and scientific discourse than domestic violence [52], p. 28. However, this might be a fallacy: E.g. Kitzmann and colleagues [37] could show equally grave effects of exposure to domestic violence and more direct abuse victimization. But even understanding domestic violence as an independent phenomenon, the findings of the current study suggest a higher child abuse potential in families with domestic violence.

### Child Abuse Risk and Use of Interventions

The final analysis aimed to answer the question, if there is a link between child abuse potential and the use of early childhood intervention. Comparing the use of universal and targeted prevention services resulted in a nuanced picture. Universal prevention services, e.g. parent–child courses, were more frequently used by families with low child abuse potential. These services are usually available for a fee only, and require knowledge, obviously about their existence, but also about their specific requirements. Families with multiple burdens may thus face significant barriers to accessing such services.

In contrast with universal prevention services, parents with higher abuse potential used targeted prevention services more often than parents with lower risk. This shows that targeted services like child counseling, home visits and child protection services seem to be successful in guidance reaching target groups and motivating them to participate in early childhood intervention. However, the results of the current study hint at a gap in the provision of targeted prevention, which are meant to reach families in need: In general, the use of targeted prevention is rare. Although families with higher abuse potential used targeted prevention services more often, there were still 7.4% (n = 13) of the families at T2 with high abuse risk (> 9) who were not involved in any targeted prevention. As such, the data indicate that the system of early childhood intervention still does not satisfactorily reach all at-risk families at an early stage. Implementing early screening of families at risk might be one solution to scale down this shortcoming.

### Usefulness of BCAP for Risk Screening in Research

How to assess the precursors of child maltreatment is part of an ongoing discussion. Although not in the main focus of the current study, the findings of this study indicate that, overall, BCAP is a useful instrument for risk assessment. Due to the questionnaire’s brevity, it proves to be highly economical and may also be used for screening purposes in large-scale samples. Furthermore, it has been shown that the short form of the CAP offers similar results to the long form [22], and that the factorial structure of the BCAP could be validated in German populations as well—at least for mothers [24]. The moderate to strong associations with various risk factors in the current study, which were linked to actual child abuse according to other studies [9], provide further proof for the construct validity of the BCAP in assessing abuse risk.

However, analyses showed that high percentages of BCAP protocols were invalid according to established criteria [23]. These results are similar to other studies in the U.S. and the U.K. [22, 23]. For the purpose of the current study, we choose to include the invalid protocols while simultaneously confirming each result for valid protocols only. Our results give no proof for the difference between both groups. Rather, excluding invalid protocols would have contracted almost one third of the sample, a sample with more risk factors. It appears that the established exclusion criteria might be too restrictive–at least in our study. More research is needed to test the feasibility of the validity scale itself in order to give more robust indications how to deal with potential difficulties.

## Strengths, Limitations, and Future Directions

To our knowledge, the KiD 0-3 in-depth study was the first study using the BCAP in Continental Europe. This study allowed for a direct comparison of single or specific combinations of risk factors with a cumulative risk model, and to combine data on child abuse potential with parent-reported family violence and use of early childhood intervention. Because we aimed to explore families with different levels of risk, we benefited considerably from the sample selection. For the in-depth study, we succeeded in recruiting groups, which are usually difficult to reach (e.g. those with immigrant background, low level of education, etc.). We could thus successfully analyze this heterogeneous sample of young parents with a variety of risk factors in a longitudinal design.

Despite these strengths, the present study is also subject to a number of limitations. Due to the large amount of collected data [27], the sample is rather small (*N* < 200), and some risk factors were present in only a few families. Also, we could only rely on self-report data for the current purposes, in part with only brief indicators suitable as a screening measure at most. Moreover, self-reports may present a biased picture compared to observations or objective data, particularly regarding the assessment of sensitive data like actual child abuse and the potential for such maltreatment. From other studies it is clear that single informant reports lead to an underestimation of maltreatment [17, 18]. Thus, our findings need to be interpreted on the basis of these limitations. At the same time, to gain insight into a phenomenon which mostly occurs inside the family, and in the absence of the involvement of child protection services, we necessarily had to rely on self-report data in order access information. If available we used widespread and internationally validated instruments.

Furthermore, the current study tended to confine itself to the assessment of risk factors instead of also looking at protective factors. Just as an accumulation of risk factors might have negative consequences, cumulating protective factors can lead to positive outcomes [53] or have compensatory effects. Therefore, future studies should examine whether protective factors could buffer the negative consequences of cumulative risk.

The findings of our study indicate that the BCAP is an economical instrument for measuring child abuse risk in survey research, significantly associated with a variety of relevant risk factors. As the brief indicator of parental anger used here was an important predictor for child abuse risk, future studies should test whether including a more extended, and so, more accurate indicator of parental anger could improve the BCAP’s predictive power of the risk for child maltreatment. In addition, research should focus on associations between the BCAP and official statistics as well as indices of child development in order to validate the BCAP as effective assessment method, but also to gain insights into which parents are overlooked when using self-reported child abuse risk compared with actual incidents. Further research should address the composition and usefulness of the BCAP validity scale.

## Summary

To protect children from harm, the early identification of families with a (high) risk for child maltreatment is an important goal. Since self-reports and official statistics generally underestimate the prevalence of child abuse, for prevention purposes it is particularly important to identify the accumulating risks and precursors of actual maltreatment. Accordingly, this study focused on various risk factors for child maltreatment and the longitudinal links between abuse risk and caregiver-reported abuse occurrence. Subsequently, the associations between abuse risk and involvement in early intervention were investigated. Data of 197 German caregivers with children under 3 years of age were collected in two waves. In addition to employing the Brief Child Abuse Potential Inventory (BCAP) to assess abuse risk, socio-demographic, parent, child and family-related risk factors were assessed using screening tools.

Maternal psychopathology and anger, parenting stress, and maternal experiences of maltreatment in childhood were associated with abuse risk at T1. Longitudinal changes in BCAP inventory abuse risk were linked to caregiver’s level of education and child-related factors. Interestingly, the accumulation of risk factors did not explain more variance than distinct risk factors combined. Furthermore, highly significant associations were found between BCAP and caregiver-reported abuse. However, although families with higher abuse potential used targeted prevention services more often, some burdened families could not be reached by early childhood intervention. Using the BCAP as a screening tool might be helpful to improve referral to adequate interventions.
